# Editorial: Tuberculosis in domestic ruminants: towards eradication of zoonotic tuberculosis

**DOI:** 10.3389/fvets.2024.1453876

**Published:** 2024-07-04

**Authors:** Maria Laura Boschiroli, Francisco Javier Salguero, Gareth Jones, Javier Bezos

**Affiliations:** ^1^Agence Nationale de Sécurité Sanitaire de l'Alimentation, de l'Environnement et du Travail (ANSES), Maisons-Alfort, France; ^2^UK Health Security Agency (UKHSA), London, United Kingdom; ^3^Animal and Plant Health Agency, Addlestone, United Kingdom; ^4^Department of Animal Health, Faculty of Veterinary Medicine, Complutense University of Madrid, Madrid, Spain; ^5^VISAVET Health Surveillance Centre, Complutense University of Madrid, Madrid, Spain

**Keywords:** tuberculosis, ruminants, eradication, epidemiology, diagnosis, control

## Introduction

Tuberculosis (TB) is a zoonosis with a significant impact on human and animal health and it remains endemic in many countries around the world. Most cases of human TB are caused by *Mycobacterium tuberculosis*, whilst TB in wildlife and domestic ruminants is mainly caused by animal adapted mycobacterial species such as *M. bovis* and *M. caprae*. However, the impact of zoonotic TB (zTB) has been known for a long time and resulted in the implementation of milk pasteurization as a preventive public health measure in many countries. According to data published by the WHO, more than 140,000 people are infected and more than 12,000 die every year due to zTB, particularly in the African and Southeast Asian regions. Zoonotic *M. bovis* transmission represents 0.4% of all cases in the EU, although this may be underestimated, as it is not always possible to confirm the TB species involved, or conduct appropriate epidemiological investigations. Livestock TB control programs were originally implemented to reduce zTB. Successes in controlling infections in livestock, together with measures to control animal products with suspected infection have contributed to lowering the annual incidence of cases of zTB in high-income countries. The incidence tends to be higher in countries where animal and public health resources are inadequate to manage costly bovine TB control programs. TB eradication is difficult to achieve mainly due to: (I) limited knowledge of the complex immunological response against the infection, (II) complex epidemiology, including several domestic and wildlife reservoirs, (III) the absence of an effective vaccine, (IV) a limited performance of the current diagnostic tests, and (V) societal aspects. Therefore, there is a need to continue working on these fields of TB research. In this Research Topic, different aspects of the epidemiology, diagnosis and control of TB in wildlife and domestic ruminants have been addressed to increase the knowledge about this important zoonotic disease. This will contribute to TB eradication in domestic ruminants, which also represents an important step to reduce the zTB.

## Epidemiology

Cattle have been traditionally considered the main animal reservoir of TB and the primary cause of zTB. However, as described in studies from this Research Topic, other ruminants such as sheep (Juste et al.), goats (Velasco et al.), water buffalo (Martucciello et al.), and deer (Li et al.) are susceptible to TB and can play an important role in the epidemiology of the disease, either due to the high prevalence or the epidemiological link they may have with other animal species and humans. This indicates the need for comprehensive control and eradication strategies, that should take all animal reservoirs into account, and not just cattle. This is challenging due to the difficulty of diagnosing TB in certain animal species and the high cost. Diagnosis is often unaffordable, especially in middle- or low-income countries where zTB cases are much more frequent due to the limitations in implementing hygienic-sanitary measures and the closer contact that often exists between humans and animals in rural communities, as described in the study of Didi et al. The authors highlight the need for strong coordination between policymakers, public health, and veterinary services and civil society organizations to implement targeted sensitization and awareness campaigns for communities with less knowledge and attitudes regarding TB. Moreover, in relation to the different susceptibility of animal species to TB, the findings of Juste et al. are very interesting. They observed a high sensitivity of sheep to infection by a specific strain of *M. bovis* (SB2737), challenging the previously attributed high natural resistance to infection in this species and suggesting an important role as a TB reservoir in certain epidemiological contexts.

## Diagnosis

Diagnosis is one of the main pillars of TB control and eradication programs in ruminants and poses a significant challenge, which is directly related to the persistence of outbreaks and difficulties in eradication. In both domestic and wild ruminants, the gold standard *ante-mortem* diagnostic test is the intradermal tuberculin test, which is not perfect in terms of sensitivity and specificity. In certain situations, it is used in parallel with the interferon-gamma release assay (IGRA) to maximize the detection of infected animals. The use of IGRA has been primarily focused on cattle, although its application in other domestic ruminants has also significantly contributed to reducing the prevalence of the disease, as described by Martucciello et al. in water buffaloes. A direct blood PCR based on IS6110 target has been also evaluated by Encinas et al. in cattle, showing usefulness to maximize the diagnostic sensitivity in a subset of animals showing a high humoral response. Due to the limited performance of the official tests, ongoing research to improve TB diagnosis is required. In this regard, antibody-based detection assays stand out as potential alternative tools with a high cost-benefit ratio, although their sensitivity has been generally reported as low in cattle. However, their performance seems much better in other ruminants not subjected to compulsory eradication programs, suggesting that antibody-based detection assays could be a valuable diagnostic tool not only in sheep (Juste et al.) but also in goats (Velasco et al.), cervids (Li et al.), or water buffalo, a species in which its potential use would be valuable to accelerate TB eradication (Martucciello et al.).

## Control

The difficulties in eradicating TB in humans and animals are largely due to the absence of an effective vaccine. Currently, the only available vaccine is the BCG vaccine, which is a century old. This vaccine has been used experimentally in animals, mainly domestic ruminants, and demonstrates variable efficacy. Vaccination does not completely prevent infection but reduces susceptibility to it and the severity of TB pathology, which is thought to correlate with lower transmissibility. Based on these findings, some studies have suggested that implementing BCG vaccination could improve the control of the disease in countries without the capacity to implement programs based on test and slaughter strategies, or to accelerate the eradication goal in regions where such programs are already in place. Interestingly, the BCG vaccine can also induce non-specific effects on the immune system, enhancing responses to infections caused by unrelated pathogens, and having non-specific effects on lactation, as demonstrated by Contreras et al. who report that these effects may be influenced by the animal breed and season.

## Author contributions

MB: Writing – original draft, Writing – review & editing. FS: Writing – original draft, Writing – review & editing. GJ: Writing – original draft, Writing – review & editing. JB: Conceptualization, Supervision, Writing – original draft, Writing – review & editing.

